# Use of Mpox Multiplex Serology in the Identification of Cases and Outbreak Investigations in the Democratic Republic of the Congo (DRC)

**DOI:** 10.3390/pathogens12070916

**Published:** 2023-07-07

**Authors:** Eddy Kinganda-Lusamaki, Lionel Kinzonzi Baketana, Etienne Ndomba-Mukanya, Julie Bouillin, Guillaume Thaurignac, Adrienne Amuri Aziza, Gradi Luakanda-Ndelemo, Nicolas Fernandez Nuñez, Thierry Kalonji-Mukendi, Elisabeth Simbu Pukuta, Antoine Nkuba-Ndaye, Emmanuel Lokilo Lofiko, Emile Malembi Kibungu, Robert Shongo Lushima, Ahidjo Ayouba, Placide Mbala-Kingebeni, Jean-Jacques Muyembe-Tamfum, Eric Delaporte, Martine Peeters, Steve Ahuka-Mundeke

**Affiliations:** 1 TransVIHMI, University of Montpellier (UM), French Institute of Health and Medical Research (INSERM), French National Research Institute for Sustainable Development (IRD), 34394 Montpellier, France; 2 Institut National de Recherche Biomédicale (INRB), Kinshasa P.O. Box 1197, Democratic Republic of the Congo; 3 Service de Microbiologie, Département de Biologie Médicale, Cliniques Universitaires de Kinshasa (CUK), Université de Kinshasa (UNIKIN), Kinshasa P.O. Box 127, Democratic Republic of the Congo; 4 Programme National de Lutte Contre le Monkeypox et les Fièvres Hémorragiques Virales, Ministère de la Santé (PNLMPX-FHV), Kinshasa P.O. Box 1197, Democratic Republic of the Congo

**Keywords:** Mpox, multiplex serology, outbreak, Democratic Republic of the Congo

## Abstract

Human Mpox cases are increasingly reported in Africa, with the highest burden in the Democratic Republic of Congo (DRC). While case reporting on a clinical basis can overestimate infection rates, laboratory confirmation by PCR can underestimate them, especially on suboptimal samples like blood, commonly used in DRC. Here we used a Luminex-based assay to evaluate whether antibody testing can be complementary to confirm cases and to identify human transmission chains during outbreak investigations. We used left-over blood samples from 463 patients, collected during 174 outbreaks between 2013 and 2022, with corresponding Mpox and VZV PCR results. In total, 157 (33.9%) samples were orthopox-PCR positive and classified as Mpox+; 124 (26.8%) had antibodies to at least one of the three Mpox peptides. The proportion of antibody positive samples was significantly higher in Mpox positive samples (36.9%) versus negative (21.6%) (*p* < 0.001). By combining PCR and serology, 66 additional patients were identified, leading to an Mpox infection rate of 48.2% (223/463) versus 33.9% when only PCR positivity is considered. Mpox infections were as such identified in 14 additional health zones and 23 additional outbreaks (111/174 (63.8%*)* versus 88/174 (50.6%)). Our findings highlight the urgent need of rapid on-site diagnostics to circumvent Mpox spread.

## 1. Introduction

Mpox, previously called Monkeypox (MPX), is a zoonosis caused by the Monkeypox virus (MPXV) from the *Orthopox* genus. Mpox occurs mainly in central and west Africa and causes a disease with symptoms similar to those previously observed in smallpox patients [1,2,3]. The first case of Mpox in humans was identified in 1970 in the Democratic Republic of the Congo (DRC) [4]. With the eradication of smallpox in 1980, human Mpox cases have increasingly been reported in central and west Africa. Before 2022, 11 African countries have reported human cases of Mpox, i.e., Benin, Cameroon, the Central African Republic (CAR), Cote d’Ivoire, DRC, Gabon, Liberia, Nigeria, the Republic of the Congo (RC), Sierra Leone and South Sudan [5,6,7]. The highest burden of the disease is reported in DRC, where more than 90% of Mpox cases occurred. Nevertheless, sporadic cases have been documented outside Africa, but always with an epidemiologic link to the continent [8,9]. There are also two distinct clades of MPXV viruses; clade I circulates in Central Africa (Congo Basin) and clade II in West Africa, with the latter clade being less pathogenic [1,5,9,10].

Mpox has been considered as a rare and neglected disease, but the recent large outbreak that started in May 2022 and infected more than 80,000 individuals in more than 100 countries over the world highlighted the global public health threat of Mpox [11,12]. Mpox outbreaks are supposed to be the result of spillover events from animals to humans, and subsequent human-to-human transmission chains are limited to a few individuals [7,13]. But the recent global outbreak, which was caused by a strain close to one from an outbreak in 2017 in Nigeria [9,11,13], suggests that human transmission has been ongoing for a longer period than previously thought. The new strains have been classified as clade IIb or III depending on the authors [14,15]. The true burden of Mpox in Africa is not known and it cannot be excluded that human transmission and pauci-or asymptomatic infections are underestimated in Africa. 

In DRC, the highest affected country in Africa, Mpox is one of 21 reportable diseases or health events in the national Integrated Disease Surveillance and Response (IDSR) program. The majority of Mpox cases occur in remote areas and case reporting is thus mainly based on clinical signs according to the WHO case definition [14,16]. Mpox disease can resemble various other diseases with generalized skin eruption or rash-like measles, bacterial skin infections, scabies and chickenpox, also known as varicella [17]. The high similarities in the clinical presentation of varicella can lead to up to 50% of varicella cases that are confused with Mpox [2,18,19,20,21]. Moreover, Mpox/varicella coinfections can also occur [22]. Based on clinical signs only, there is thus likely an overestimation of Mpox cases. Since 2004, confirmatory testing by polymerase chain reaction (PCR) in DRC, is only available in the National Reference Laboratory at the “Institut National de Recherche Biomédicale” (INRB) located in Kinshasa, the capital city, but is rarely performed to confirm clinically notified case numbers. The most suitable specimens for laboratory confirmation are swabs or crusts from the skin lesions. Blood is not recommended for molecular diagnosis because of the short and variable viremic period [2,14]. However, remote health care centers are often not equipped to safely collect crusts or swab samples, and instead blood samples are collected and shipped to the reference laboratory for the large majority of cases. Underestimation of clinically confirmed cases by PCR in the reference laboratory is thus very likely, especially for patients in remote areas who often arrive with a delay after the onset of symptoms. In addition, adequate storage, processing and shipment of samples from remote areas, with equatorial climates, to the reference laboratory can also have a negative impact on PCR results. Therefore, antibody detection could be an alternative and complementary test to confirm Mpox infections. IgG antibodies can be detected soon after a rash [2]. However, the challenges with serological assays are cross-reactions with other orthopox viruses because they share many conserved surface antigens, which is especially crucial in serological assays that are based on whole virus lysates. Dubois and colleagues developed an Elisa based on a combination of conjugated peptides that are specifically for the detection of antibodies against MPXV without cross-reactivity with smallpox antibodies in vaccinated people. The test has high sensitivity and specificity to detect Mpox antibodies early in infection (2–6 months) and more than 2 years after infection [23].

Here we adapted the optimized peptide-based Elisa on a multiplex serological platform to evaluate to what extent antibody testing can be complementary to the current Mpox surveillance system in DRC and can provide additional information on eventual human transmission chains during epidemiologic investigations around the outbreaks, such as occurred recently in Masimanimba, a newly affected area in the Kwilu province in DRC.

## 2. Materials and Methods

### 2.1. Study Samples

Left-over blood samples with corresponding Mpox and/or Varicella-Zona Virus (VZV) PCR results from the national surveillance program were used for this study. Samples were from different cases and outbreaks that occurred between 2013 and 2022 across the country. Sample collection and shipment to the reference laboratory in Kinshasa for confirmation of Mpox infection was achieved in the framework of the national Integrated Disease Surveillance and Response (IDSR) program (Supplementary Figure S1) in collaboration with the National Program for the Control of Monkeypox and Viral Haemorrhagic Fevers (PNLMFH). Authorization for further analysis and use of data was obtained by the Ethical Committee of the Ecole de Santé Publique de l’Université de Kinshasa (ESP-UNIKIN, Number ESP/CE/05/2023.

Limited demographic data, like sex, age, health zone and province, as well as information on onset of symptoms were also available for the majority of the samples. All samples are from patients that meet the clinical case definition, i.e., “An acute illness with fever >38.3 °C (101 F), intense headache, lymphadenopathy, back pain, myalgia, and intense asthenia followed one to three days later by a progressively developing rash often beginning on the face (most dense) and then spreading elsewhere on the body, including soles of feet and palms of hand” [16]. Blood samples were kept at −20 °C since their arrival at INRB. Samples used in the study were those for which adequate volumes were still available.

### 2.2. PCR Detection, Differential Testing and Sequencing

We extracted DNA at INRB using a Qiagen DNA Mini kit from blood samples and subsequently screened for Mpox with an Orthopoxvirus-specific real-time PCR assay followed by a real-time PCR assay targeting the varicella-zoster virus (VZV) [24]. Orthopox (OPX)-positive samples were presumptively considered as Mpox-positive, because other known human Orthopox viruses do not cause the same clinical symptoms [14]. In order to confirm Mpox infection in the newly affected area in Masimanimba, whole genome sequencing was attempted on samples from the index case by Next Generation Sequencing (NGS). The library preparation was performed using Illumina DNA Prep with Enrichment and the libraries were enriched for Mpox using biotinylated custom probes synthesized by Twist Biosciences as described previously [25,26].

### 2.3. Bioinformatics and Phylogeny

From the fastq files obtained, a bioinformatic pipeline, GeVarLi (GEnome assembly, VARiant calling and LIneage assignation; https://forge.ird.fr/transvihmi/GeVarLi, accessed on 5 July 2023), was used to control reads quality and clean them. Cleaned reads were aligned to NC_003310.1 MPXV genome, and the viral lineage was assigned using Nextclade. Further, variants calling and genome coverage statistics were performed and a consensus genome sequence generated lead to a fasta format file. The sequence was deposited on GISAID with accession number EPI_ISL_14201642.

The newly identified MPXV sequence was aligned with other reference sequences that have been detected in humans and rodents, mostly in DRC, representing the diversity of currently known Mpox lineages. Multiple sequence alignment was performed using MAFFT v7 (https://mafft.cbrc.jp/alignment/server/, accessed on 5 July 2023 ). The best scalable model based on alignment data was obtained using the MegaX version 10.0.5 software [27]. The phylogeny was inferred by maximum likelihood (ML) using PhyML. Branch support was estimated with 100 bootstrap resampling [28]. The trees have been formatted and annotated with FigTree v1.4.4 software (http://tree.bio.ed.ac.uk/software/figtree/, accessed on 5 July 2023) and Inkscape (https://inkscape.org, accessed on 5 July 2023).

### 2.4. Antibody Testing

Recombinant proteins and peptides were used to optimize the multiplex serological assay at TransVIHMI unit at the Institut de Recherche pour le Développement (IRD) in Montpellier (France) and INRB, Kinshasa (DRC). The evaluated set included 3 commercially available recombinant proteins, which are C19L from Left terminal region (ProteoGenix SAS, Schiltigheim, France), D13L Central conserved region (ProteoGenix SAS, Schiltigheim, France) and A29 the Right terminal region (SinoBiological, Advion Interchim Scientific, Montluçon, France); and 3 synthetic peptides from Right terminal region B (B21R.179/180, B21R.185/186 and B22R.64/65), previously identified by Dubois and colleagues [23]. These peptides were synthesized as 31-mer peptides with an additional N-terminal cysteine residue (82–95% purity) and conjugated to bovine serum albumin (BSA) (Eurogentec, Seraing, Belgium). Reconstituted peptide-conjugates (2 mg/mL in H_2_O) were stored at –80 °C until use. We used our previously described protocol for coupling the conjugated peptides or recombinant proteins at different concentrations to Luminex beads to optimize the assay conditions [29]. Briefly, conjugated peptides (5 μg/1.25 × 10^6^ beads) and recombinant proteins (2 μg/1.25 × 10^6^ beads) were covalently coupled on carboxyl functionalized fluorescent magnetic beads (Luminex Corp., Austin, TX, USA) with the BioPlex amine coupling kit (Bio-Rad Laboratories, Marnes-la-Coquette, France) according to the manufacturer’s instructions. Unreacted sites were blocked with blocking buffer from the amine coupling kit. Peptide-coupled microsphere preparations were stored in storage buffer (Bio-Rad, Marnes-la-Coquette, France) at 4 °C in the dark until use. Before use, peptide-coupled beads were diluted to 2000 beads/μL of assay buffer (Phosphate Buffered Saline (PBS) containing 0.75 mol/L NaCl, 1% (*w*/*v*) bovine serum albumin (Sigma Aldrich, Saint-Quentin Fallavier, France), 5% (*v*/*v*) heat-inactivated fetal bovine serum (Gibco-Invitrogen, Cergy Pontoise, France), and 0.2% (*v*/*v*) Tween-20 (Sigma-Aldrich, Saint-Quentin Fallavier, France). Fifty microliters of bead mixture were added to each well of 96-well flat-bottom chimney plates (Greiner bio one, Frickenhausen, Germany). The recombinant proteins and peptides were evaluated on a panel of 60 samples from patients confirmed with Mpox at INRB, Kinshasa and 30 control samples from individuals in France born after 1980. Antigens with areas under curve (AUC) of the Receiver Operating Characteristics (ROC) analysis above 0.9 were kept for the screening. After optimization, patient samples were then tested at INRB with the optimized conditions; i.e., a 1/200 dilution and incubated for 2 h at 37 °C with peptide-coated beads in the dark on a plate shaker at 300 rpm/min. After washing, 50 μL of biotin-labeled anti-human IgG was added (BD-Pharmingen, Le Pont De Claix, France) at a concentration of 1 μg/mL in each well and incubated for 30 min in the dark while shaking at 300 rpm. Fifty μL of streptavidin-R-phycoerythrin (Fisher Scientific/Life Technologies, Illkirch, France) at 1 μg/mL were added per well after washing, and incubated for 10 min with shaking at 300 rpm. Magpix equipment (Bio-Rad, Marnes-la-Coquette. France) was used to read antigen-antibody reactions. At least 100 events were read for each bead set, and the results were expressed as Median Fluorescence Intensity (MFI) per 100 beads. For the calculation of cut-off values, we tested 90 Mpox negative samples from DRC collected during routine screening for Human Immunodeficiency Virus (HIV) infection. The cut-off was calculated as the mean plus three standard deviations for each peptide. On each plate, one positive and one negative known sample were included and tests were not validated when inter-assay variability was above 15%. We considered samples positive for a given antigen if they presented MFI above the cut-off value for this antigen. Samples were considered positive for Mpox infection when reactivity to at least one peptide was observed as recommended by Dubois and colleagues [23].

### 2.5. Statistical Analysis

We used the Chi-square and the non-parametric Wilcoxon Mann–Whitney tests implemented in Stata 15 software to compare proportions and medians of age, MFI values for each peptide between Mpox PCR positive and negative samples. The significance level was set at 0.05. MFI distribution were represented graphically using PRISM version 9 software.

## 3. Results

### 3.1. Assay Optimization and Antibody Testing for Mpox Outbreak Confirmation

Overall, six antigens were used to optimize the multiplex serological assay as described in Methods on a subset of 60 Mpox PCR positive samples, out of the 463 with corresponding Mpox PCR results used in this study. The three peptides (B21R.179/180, B21R.185/186 and B22R.64/65) had the best performances and were selected to be used in our panel. The areas under curve (AUC) of the Receiver Operating Characteristics (ROC) curves per protein summarizing their performances were best for the three peptides; i.e., 0.9575, 0.9506 and 0.9486 for B22R.64/65, B21R-180/186 and B21R-179/180, respectively, versus 0.8578 for C19L(A), 0.8398 for A29(B), and 0.7092 for D13L(C) (Supplementary Figures S2 and S3).

Left over blood samples from the total of 463 patients with corresponding Mpox and VZV PCR results from the national surveillance program were then used to evaluate to what extend serological testing has an additional value in Mpox outbreak confirmation. All assays were performed at the national reference laboratory at INRB, Kinshasa. Samples were from 174 different outbreaks that occurred between 2013 and 2022 in 22 of the 26 provinces of the country. Specifically, samples were collected from a total of 129 different health zones out of the 519 in DRC. The majority of analyzed samples are from 2022, representing 47.3% (219/463) of the total tested. In this same year, we registered a higher number of blood samples that have been collected and shipped to the INRB (241 samples), compared to previous years’ 41–135 samples/year as shown (Supplementary Figure S4), which also reflects the higher number of clinical suspect cases reported in 2022. 

The characteristics of patients and samples are shown in Table 1. Overall, 214 (46.2%) samples were from female patients, 229 (49.5%) from male, and for 20 (4.3%) samples no information on sex was available. The median age of patients was 12 years (IQR 5–25, range <1 to 78 years). The median time from rash onset to sample collection was known for 378 samples (81.6% of the dataset) and was 6 days (IQR, 4–10; range, 1 to 95 days). The median time of shipment of samples was 12 days (IQR, 8–20; range, 0–66 days) for 431 samples (93.1% of the dataset) with information available.

A total of 157 (33.7%) samples were positive by Orthopox PCR and thus classified as Mpox-positive, and 306 were negative for Orthopox (Table 1), among which 277 out of 305 tested were also negative for VZV PCR. Mpox PCR-positive samples were observed in 17 of the 22 provinces and in 71 (55%) of the 129 health zones from which samples were tested (maps in Supplementary Figure S5). Among the 463 samples tested, a total of 124 (26.8%) had antibodies to at least one of the three MPXV peptides. The B22R-64/65 peptide detected the highest number of seropositive samples (99/124, 79.8%), and an additional 25 (20.2%) were detected with the other peptides (Table 2). It was not possible to evaluate whether reactivity with a particular peptide was related or not to the delay between symptom onset ant testing given the low numbers of reactive samples for certain peptides.

**Figure 1:**
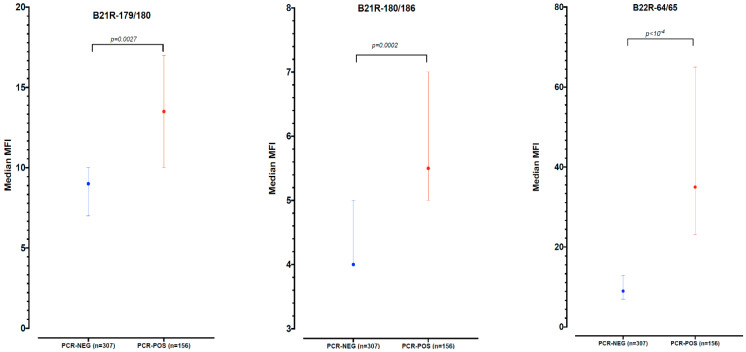
MFI distribution per peptide for Mpox PCR negative (blue) and positive (red) samples.

The proportion of antibody positive samples was higher in samples that were confirmed for Mpox infection by PCR; i.e., 58/157 (36.9%) versus 66/306 (21.6%) for the Mpox PCR negative samples with *p* < 0.001 (Table 1), regardless of the delay between symptoms and sampling. After running a Wilcoxon rank-sum test for each peptide, the median MFI values were higher in Mpox PCR-positive samples than in negative results, with *p*-values estimated to be 0.0027, 0.0002 and <0.0001 for B21R179/180, B21R180/186 and B22R64/65, respectively (Figure 1). Overall, 99 (21.4%) samples were only positive by PCR, 58 (12.5%) were positive by PCR and serology and 66 (14.3%) were positive only by serology. This means that by combining PCR and serology, an additional 66 patients were identified as infected, bringing the total Mpox infection rate to 48.2% (223/463) instead of 33.9% when only PCR positivity is considered. In our sample set, this also means that Mpox infection was confirmed in 14 additional health zones, including in urban health zones such as N’djili in Kinshasa the capital city, or Kikwit-Sud in Kwilu Province (maps in Supplementary Figures S5 and S6), i.e., 86/129 (66.7%) versus 72/129 (55.8%) affected health zones.

The median age of patients with antibodies to Mpox is comparable to those with a PCR confirmed infection; i.e., a median of 14 years (IQR, 7–25; range <1–60 years) versus 12 years (IQR, 5–25; range <1–63 years) for the 120 and 154 patients with information on age available, respectively (*p* = 0.3578) (Supplementary Table S1). Similarly, there were no significant differences using the same test (*p* = 0.523), when comparing the median age of patients with PCR confirmed infection (median, 12 years; IQR, 5–25; range, <1–63 years) to the median of age of patients with negative PCR results (median, 13 years; IQR, 6–26; range, <1–76 years) as shown in Table 1. Male patients were more frequently positive, either by PCR (83/157; 53.2%) or for antibodies (66/121; 54.6%), although not significantly (*p* = 0.523). The time between the onset of symptoms and sample collection was comparable between patients that tested positive for Mpox by PCR (*n* = 130) and those that were negative (*n* = 248); median 6 days (IQR, 3–9; range; 0–28 days) versus 6 days (IQR, 4–11; range 1–95 days), respectively. Regarding serology results, the time of onset of symptoms was statistically different (*p* = 0.0016) between patients without anti-Mpox antibodies and those with, i.e., a median of 6 days (IQR, 4–9; range, 0–95 days) for the 274 seronegative samples versus 8 days (IQR, 5–11; range, 0–52 days) for the 104 seropositive samples for whom this information was available. Overall, the delay of symptom onset and detection of Mpox by PCR was shorter as for the delay for presence of antibodies; i.e., median 6 days (IQR, 3–9; range, 0–28 days) for PCR positivity versus 8 days (IQR, 5–11; range, 0–52 days) for antibody detection.

For some Mpox alerts, samples from more than one patient were shipped for confirmation and the 463 samples tested were from 174 alerts, among which 88 (50.6%) were confirmed by PCR, and an additional 23 (13.2%) were identified by serology with a total of 111 (63.8%) outbreaks confirmed for Mpox. The 23 additional alerts were confirmed by the serology of thirty-one samples out of the sixty-six samples only positive to serology, with a median of one sample per alert. Among the sixty-three remaining outbreaks that were classified as negative for Mpox, nine were confirmed as VZV by PCR. We also noted four outbreaks with co-circulation of Mpox and VZV; i.e., two Mpox PCR confirmed outbreaks were also positive for VZV, and two additional VZV outbreaks were observed in outbreaks that were only serologically identified.

### 3.2. Antibody Testing during an Outbreak Investigation in Masimanimba Health Zone, Kwilu Province

**Figure 2:**
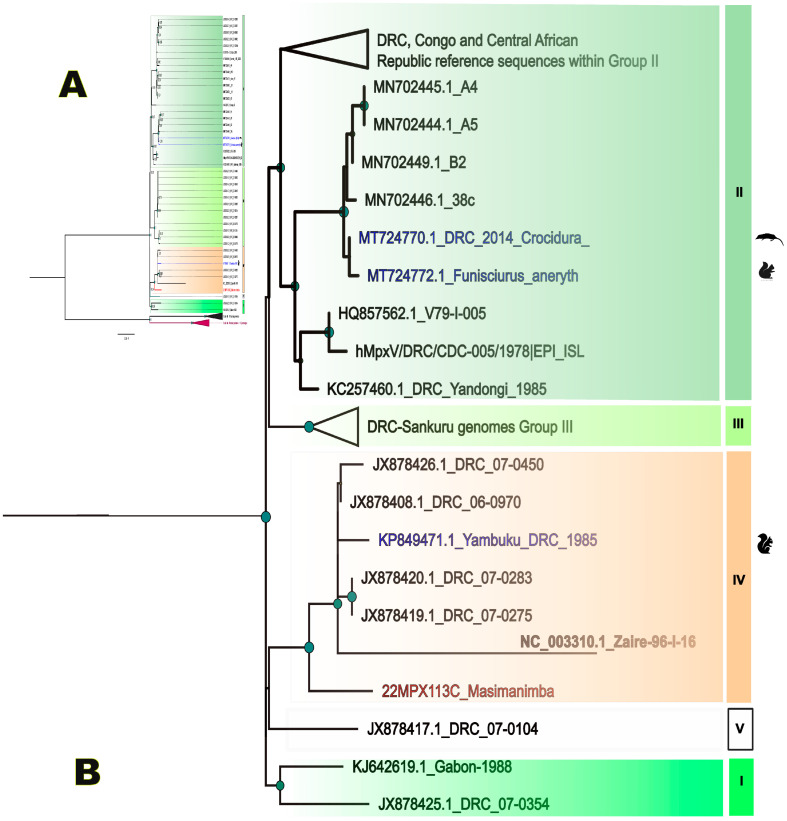
(**A**) Phylogenetic tree based on complete genomes built by maximum likelihood using the GTR+G model. Clade II sequences are used as outgroup. (**B**) Subtree highlighting the newly identified monkeypox strain in Masimanimba in red and sequences in blue are from animals. Roman numbers indicate previously described MPXV lineages [30], each MPXV lineage is highlighted in a different color.

When Mpox cases are notified in a new health zones, an epidemiological investigation is organized in the framework of the national Integrated Disease Surveillance and Response (IDSR) program. As such, the Masimanimba health zone in the Kwilu province reported a first case on the first of May 2022. The index case was a 20-year-old male student; fever and cervical lymphadenopathy started on 20 April followed by rashes 4 days later. He first received traditional care, which failed. Therefore, he was brought to the Masimanimba’s referral general hospital. The evolution of the disease and skin lesions alerted the Central Office of the Masimanimba health zone and the case was officially reported to the provincial (Kwilu province) and national health authorities on 1 May. Blood and crust samples were taken on 3 May for laboratory confirmation, and the case was confirmed positive for Mpox by universal orthopox PCR on 9 May for both samples. The Ct values were, respectively, 35.8 on blood and 17.67 on crust. Sequencing was performed on the crust sample due to its higher viral load and the whole genome sequence was obtained with 99.78% genome coverage at 3×, 94.46% at 10× and mean depth 55. Phylogenetic analysis confirmed that the strain (in red on the tree) belonged to Clade I, subgroup IV (Figure 2).

Epidemiological investigation in the field was conducted between 17 and 24 May 2022. This investigation revealed that the index case, at the basis of the national alert, is probably not the primary case of Mpox infection in the village without excluding the zoonotic source. A 25-year-old male, a friend of the index case, reported that he had similar clinical symptoms, which started 3 weeks earlier on 4 April. He was assisted by the index case during his symptomatic phase, who provided him traditional care using medicinal plants, treated as having chickenpox. Blood was drawn from this individual and serological testing revealed the presence of antibodies to Mpox peptides, suggesting that this person could be at the origin of the contamination of the case reported to the health authorities (Supplementary Figure S7).

Moreover, the epidemiological investigations also reported a case of another potential Mpox case, i.e., a man of more than 60 years old who died on December 2021 from a disease with similar symptoms. These observations illustrate that Mpox cases can go unrecognized and suggests underreporting.

## 4. Discussion

In this study, we evaluated whether testing for IgG antibodies can provide additional information during the investigation of Mpox outbreaks and identification of cases, in the framework of the national Integrated Disease Surveillance and Response (IDSR) program in the Democratic Republic of Congo (DRC). In DRC, due to shortage of collection kits, blood samples are generally used instead of crust or skin lesions, which are more appropriate for confirmation of clinical suspect cases, and it is thus very likely that PCR confirmation underestimates the number of Mpox cases. Mpox occurs mainly in remote areas, especially in Central Africa, and patients often attend the health care centers at a late stage in the disease, which could be after the viremic peak in blood. Similarly as for Mpox, crust and skin samples are more suitable than blood to diagnose infection with varicella/chickenpox (VZV), which is often clinically confounded with Mpox [14]. Therefore, serological testing may be useful when the clinical presentation and epidemiology suggest Mpox infection despite negative PCR results and in a low-resource context. In addition, instead of using each antigen separately, or more stringent criteria like reactivity to a minimum of two antigens, we chose to use positivity to at least one antigen to determine positivity and to gain sensitivity as those peptides were already shown to be specific and as recommended by Dubois and colleagues [23]. Among the 463 blood samples tested in our study from 174 clinically confirmed Mpox alerts in DRC between 2013 and 2022, 50% (87/174) were confirmed by PCR and most negative samples for Mpox also tested negative for varicella/chickenpox by PCR. Testing for MPXV-specific antibodies identified 66 additional patient samples positive for Mpox, thus increasing the positivity rate from 33.7% by PCR to 48.2% when both PCR and/or seropositivity are considered. Mpox was as such identified in 14 additional health zones in the country and in 24 additional Mpox alerts leading to a total of 63.8% alerts confirmed as Mpox. 

Overall, for almost half of the patients with clinical confirmed Mpox infection, the samples shipped for laboratory confirmation were negative by PCR and/or antibody testing for Mpox and the large majority were also negative for varicella PCR. For a proportion of these samples, PCR negativity can be due to the fact that diagnosis is performed on blood after the viremic peak for both infections. In addition, suboptimal shipping and storage conditions on site can also play a role in PCR negativity. The median time of sample shipment was 14 days, often in suboptimal equatorial climate temperature conditions that can affect both antibody and molecular detection, for example delays of 2 months were observed for some samples in our study. These logistical factors combined to the use of long storage of samples could impact sample integrity and assay sensitivity. This is especially the case for IgM antibodies that are more temperature sensitive and therefore we used IgG detection in our assay. 

The low positivity rate of antibody detection suggests that, in this early stage of the disease, IgG antibody levels are still low and that the period of IgG antibody development can be variable and longer than a few days only after the onset of rash as previously reported [2,17,31]. Detection of total immunoglobulins using anti-kappa light chain, instead of anti-IgG, did not improve the detection rate (data not shown). In our study, the median time of onset of symptoms and sample collection was 6 days. In general, antibody detection occurs later than viral detection in blood. In this sample set from the national surveillance program, the delay of Mpox detection by PCR was shorter as the delay for the presence of antibodies; i.e., a median of 6 days for PCR positivity versus 8 days for antibody detection. Although the information on symptom onset has to be taken with consideration because of the possible variability in the interpretation of symptoms in the different healthcare centers across the country and can vary significantly on the experience and training of health care personnel. Other studies in other areas from DRC or in the Central African Republic also observed that a significant proportion of clinical suspect Mpox were not confirmed [32,33]. Despite the fact that a significant proportion of samples could be false negatives by PCR and antibody detection because of the sensitivity of the assay or the short viremic period and variable period of IgG appearance, the storage conditions on site and suboptimal shipment, it cannot be excluded that, for a subset of the samples that are negative for Mpox PCR and serology as well as negative for varicella PCR, another etiologic agent is responsible for the rash. Even though the set of peptides used for serology was the best option according to the literature for specificity, it cannot be excluded that the sensitivity is too low. Today, only limited data are available on antibody dynamics in Mpox infections early in infection as well as on their long-term duration and more studies are urgently needed.

When Mpox cases are reported in new health zones in DRC, an epidemiologic investigation in the field around the new cases is organized in the framework of the national Integrated Disease Surveillance and Response (IDSR) program. This was the case in Masimanimba in May 2022, where the investigation completed with serological testing revealed that the index case was not the primary Mpox patient in this area, i.e., the first case after the zoonotic transmission event. The index case most likely acquired the disease by human to human transmission instead of a zoonotic transmission event. Moreover, clinical suspect cases seemed to be present in the area since the end of December 2021. The index case that alerted the health system was confirmed for Mpox infection by PCR on blood and crusts and the complete genome from the virus was sequenced, confirming infection with a clade I variant from Central Africa. Therefore, it is also very likely that the serological test from the contact person corresponds to specific Mpox antibodies. This patient did not attend the health-care center, but used traditional medicine and was therefore missed by the national surveillance system.

## 5. Conclusions

In conclusion, our study clearly shows that the extent of Mpox infections is not known in DRC and most likely also in other African countries after more than 50 years of occurrence and despite the threat it represents. On one hand, case reporting on clinical case definitions can overestimate the number of infections and on the other hand, laboratory confirmation by PCR only and logistical challenges can underestimate infection rates. Moreover, Mpox cases that alert the health system can already be secondary cases and are thus not always the index cases who acquired Mpox after zoonotic transmissions and therefore some Mpox alerts from the same areas over several months interval could represent human transmission chains that can be longer than initially expected. It can thus also not be excluded that certain outbreaks go unrecognized. There is thus an urgent need for rapid and point of care tests that can be used in remote areas to rapidly diagnose Mpox cases. Seroprevalence studies to evaluate the extent of Mpox infections in endemic areas and countries are also urgently needed as well as genetic characterization of Mpox virus strains in humans but also in wildlife to evaluate the proportion of human and zoonotic transmissions. The recent large outbreak of Mpox outside Africa has highlighted the global public health threat of this infection.

## Figures and Tables

**Table 1:** Characteristics of patients and samples included in this study.

Variable	Mpox PCR Positive *N* = 157 *	Mpox PCR Negative *N* = 306	Total *N* = 463	*p*
Age ^1^, years				
median (IQR ^#^)	12 (5–25)	13 (6–26)	13 (5–25)	0.523
Sex ^2^,				0.434
Female	70 (44.9%)	145 (47.2%)	214 (46.2%)	
Male	83 (53.2%)	146 (47.6%)	229 (49.5%)	
*Missing*	4 (2.6%)	16 (5.2%)	20 (4.3%)	
Delay symptoms and sampling ^3^				
Days (IQR)	6 (3–9)	6 (4–11)	6 (4–10)	0.042
Range	0–28	0–95	0–95	
Delay sample shipment ^4^				
Days (IQR)	13 (8–21)	12 (7–20)	12 (8–20)	0.435
Range	2–58	0–66	0–66	
Mpox IgG antibodies	58 (36.9%)	66(21.6%)	124 (26.8%)	<0.001 **
VZV PCR positive ^5^	0/20 (0.0%)	28/305 (9.2%)	28/325 (8.6%)	

^1^ Age is documented for 440 patients (154 Mpox PCR-positive and 286 negative). ^2^ Sex is documented for 443 patients (153 Mpox PCR-positive and 290 negative). ^3^ Delay between symptoms and sampling are documented for 378 patients (130 Mpox PCR-positive and 248 negative). ^4^ Delay of sample shipment is documented for 431 patients (155 Mpox PCR-positive and 276 negative). ^5^ VZV PCR results were available for 325 patients (20 Mpox PCR-positive and 305 Mpox PCR-negative). The VZV PCR was not performed for 137 Mpox positive patients. * Out of those 157 Mpox positive samples, 1 blood sample was negative but the vesicle from the same patient was positive. ** IgG antibody detection proportion is significantly higher in Mpox PCR positive than negative (Chi-2 test). **^#^** IQR; interquartile range.

**Table 2:** Serological results against the different peptides and peptide combinations.

Reactive Antigen(s)	Positive Samples (N = 463)	Median Days of Delay between Symptoms and Sample Collection (IQR)
B21R-179/180 only	17 (3.7%)	9.5 (5–18)
B21R-180/186 only	5 (1.1%	4 (2–6)
B22R-64/65 only	70 (15.1%)	8 (5.5–10)
Both B21R-179/180 and B21R-180/186	3 (0.6%)	2 (0–13)
Both B21R-179/180 and B22R-64/65	24 (5.2%)	9.5 (6–10.5)
Both B21R-180/186 and B22R-64/65	4 (0.9%)	37.5 (23–52)
All 3 peptides	1 (0.2%)	8
All positive to B21R-179/180	45 (9.7%)	8.5 (5–11)
All positive to B21R-180/186	13 (2.8%)	6 (2–13)
All positive to B22R-64/65	99 (21.4%)	8 (6–10)
Positive to at least one antigen	124 (26.8%)	8 (5–11)

## Data Availability

The novel sequence is available on Gisaid epipox platform with accession number EPI_ISL_14201642 (https://www.epicov.org/epi3/frontend#496aeb, accessed 6 July 2023). Serological data are available on request from the corresponding author and senior authors.

## Supplementary Materials

The following supporting information can be downloaded at: https://www.mdpi.com/article/10.3390/pathogens12070916/s1, Figure S1: Workflow of the Mpox Surveillance Algorithm in DRC, Figure S2: ROC curves summarizing peptide validation used in this study: ROC curve analysis was performed on a panel of 90 samples (60 Mpox cases from the DRC and 30 control samples from France). Figure S3: ROC curves summarizing recombinant protein validation discarded in this study: ROC curve analysis was performed on a panel of 90 samples (60 Mpox cases from the DRC and 30 control samples from France). Figure S4: Graph bars showing the percentage of samples analyzed in this study (dark grey bar) of the total number of samples tested for Mpox per year at INRB (light grey bar). The absolute number of samples included in the study or received at the INRB each year is displayed in the dark grey area and light grey area, respectively. The numbers displayed in the blue areas (upper graph) or yellow areas (lower graph) correspond to the total number of positive samples analyzed each year by PCR or serology, respectively. Figure S5: Map of DRC Map showing the number of clinical Mpox suspected samples tested by province and proportions of samples confirmed by PCR only (yellow), serology only (blue) and samples positive by serology and PCR (green). Figure S6: DRC map representing health zones in which outbreaks were not detected by PCR with serological evidence of Mpox circulation (yellow) and health zones with Mpox positive cases by PCR (yellow filled arrow head) or Serology (red asterisk fill), Figure S7: Masimanimba Health zone in the Kwilu Province and main events around the first Mpox confirmed case. Table S1: Characteristics of patients and samples per serological results.
