# Supplementary material for: Use of Mpox Multiplex Serology in the Identification of Cases and Outbreak Investigations in the Democratic Republic of the Congo (DRC)
Source: Pathogens. 2023 Jul 7;12(7):916. doi: 10.3390/pathogens12070916 (PMC10385798; doi:10.3390/pathogens12070916)
Supplement: Supplementary file 1 [file pathogens-12-00916-s001.zip › supplementary_figures_review/Figure S2.pdf]

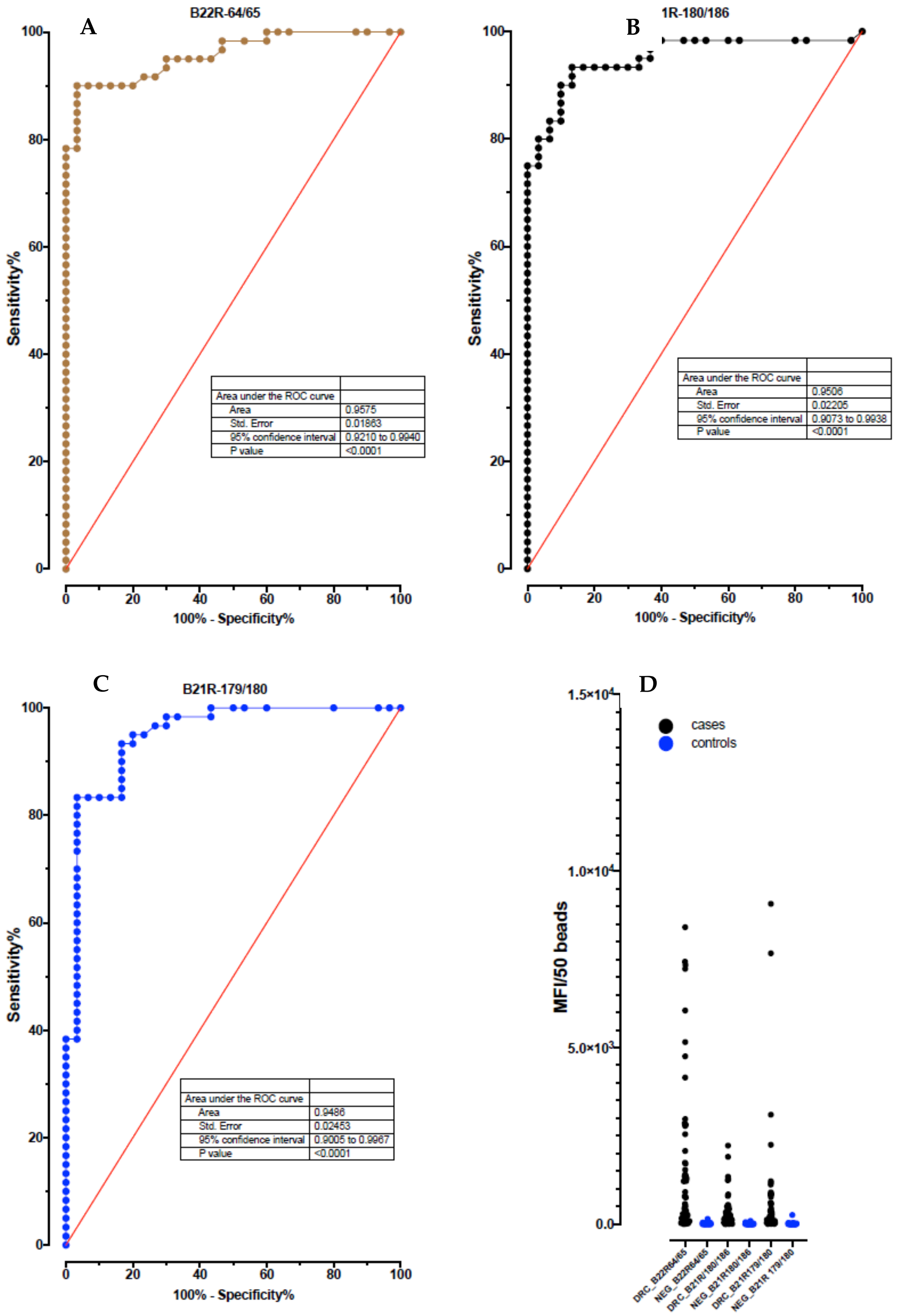

Figure S1 : Areas Under curve(AUC) of the Receiver Operating Characteristics (ROC) curves per peptid summarizing the performances of the assay for each peptid. Areas are respectively 0.9575, 0.9506 and 0.9486 for B22R.64/65 (A), B21R-180/186(B) and B21R-179/180(C). These peptids were kept for the multiplex serological assay. (D) The dotplot highlights Median Fluorecence Intensity (MFI)values per 50 beads for the panel of 90 blood samples including 60 Mpox cases (black dots) collected in the Democratic Republic of the Congo (DRC) and 30 control samples (blue dots) collected in France born after 1980). MFI are presented for each peptid and stratified by cases and controls samples. The ROC analysis was performed with GraphPad Prism 8.4.3 for MacOS.
