# Supplementary material for: Use of Mpox Multiplex Serology in the Identification of Cases and Outbreak Investigations in the Democratic Republic of the Congo (DRC)
Source: Pathogens. 2023 Jul 7;12(7):916. doi: 10.3390/pathogens12070916 (PMC10385798; doi:10.3390/pathogens12070916)
Supplement: Supplementary file 1 [file pathogens-12-00916-s001.zip › supplementary_figures_review/Figure S3.pdf]

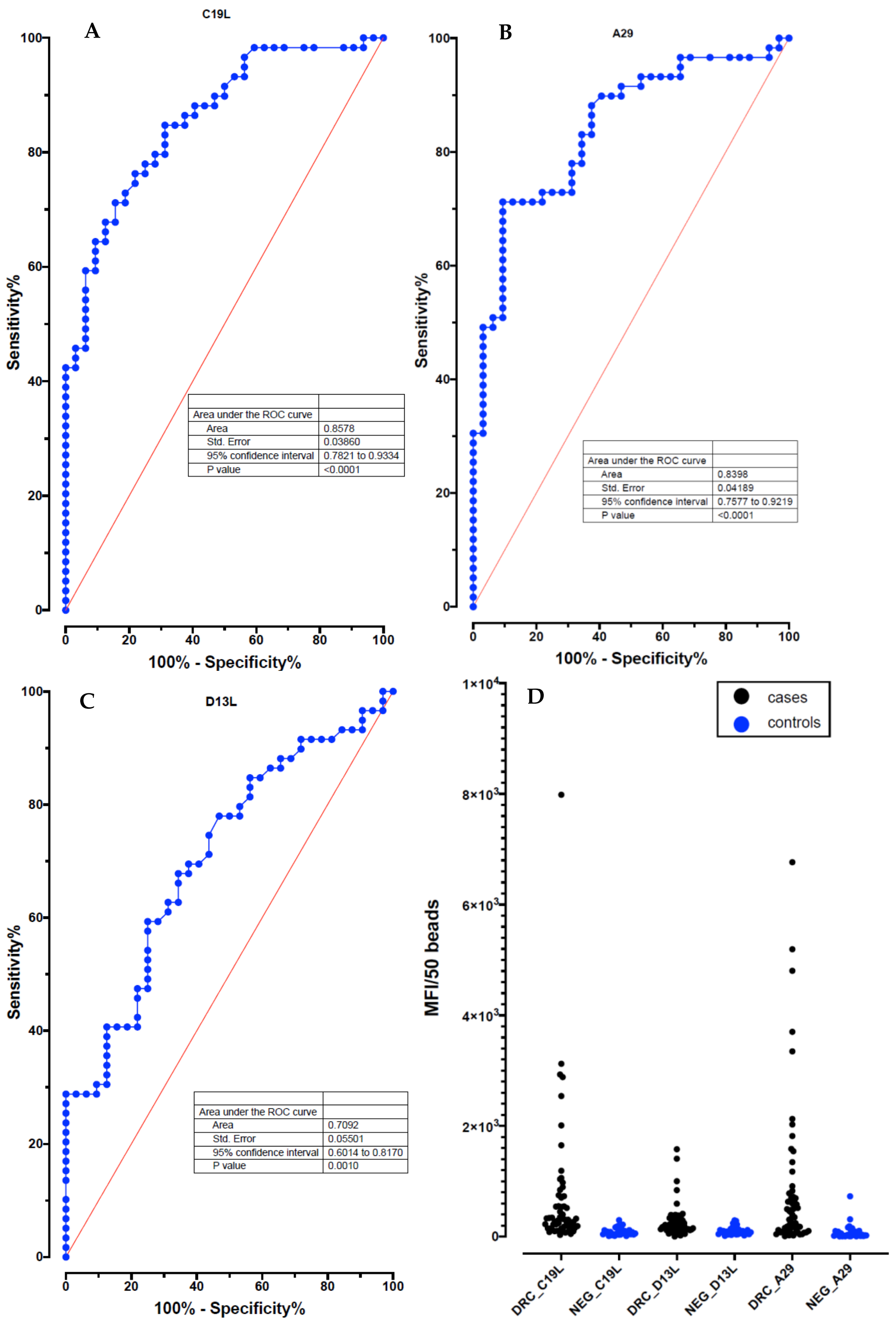

Figure S1 : Areas Under curve(AUC) of the Receiver Operating Characteristics (ROC) curves per protein summarizing their performances. AUC are 0.8578 for C19L(A), 0.8398 for A29(B), 0.7092 for D13L(C). Dots plots (D) summarize MFI/50 beads for the panel of 90 blood samples including 60 Mpox cases (black dots) collected in DRC and 30 control samples (blue dots) collected in France born after 1980. MFI are presented for each protein and stratified by cases and controls samples. The ROC analysis was performed with GraphPad Prism 8.4.3 for MacOS.
