# Supplementary material for: Use of Mpox Multiplex Serology in the Identification of Cases and Outbreak Investigations in the Democratic Republic of the Congo (DRC)
Source: Pathogens. 2023 Jul 7;12(7):916. doi: 10.3390/pathogens12070916 (PMC10385798; doi:10.3390/pathogens12070916)
Supplement: Supplementary file 1 [file pathogens-12-00916-s001.zip › supplementary_figures_review/Figure S4.pdf]

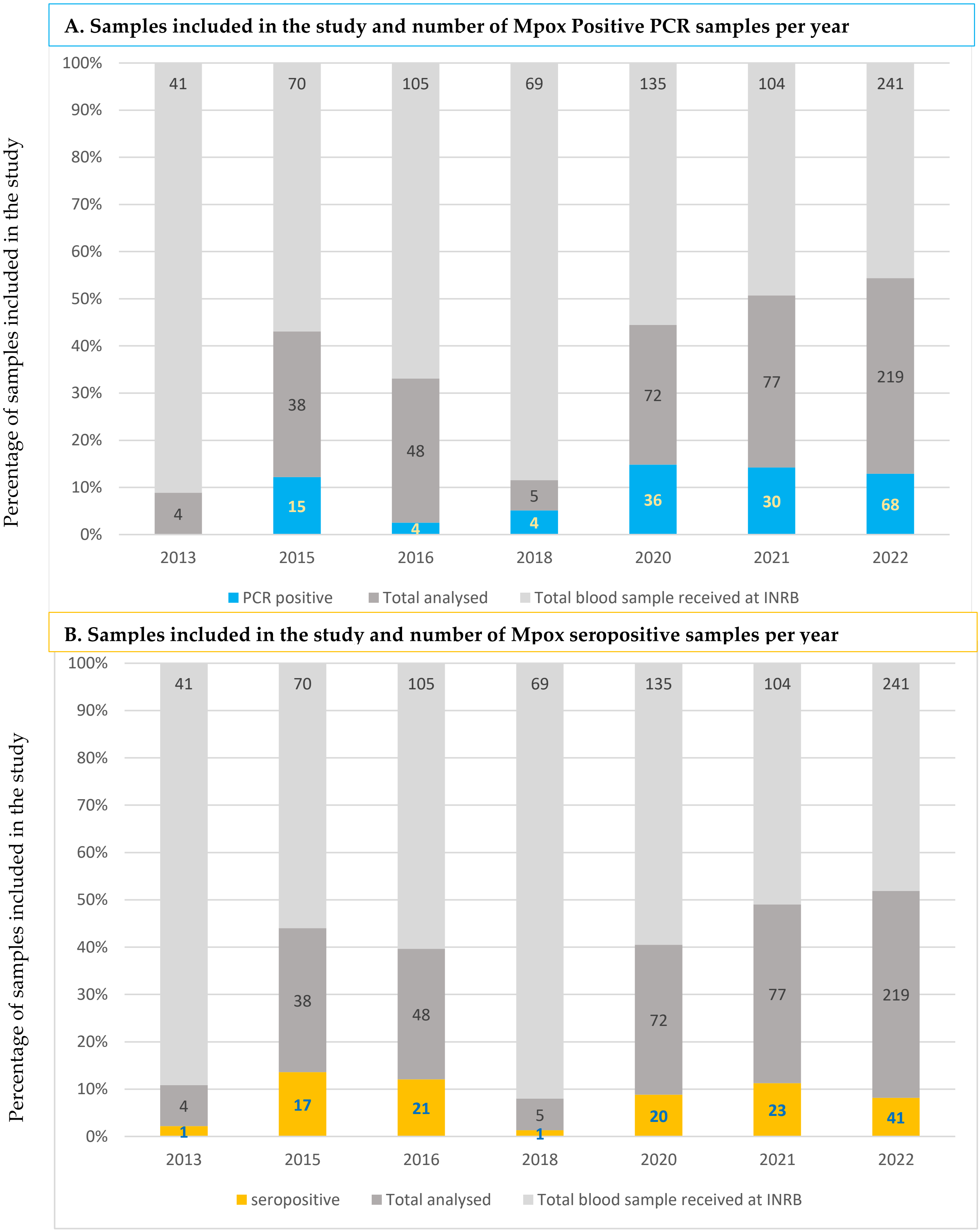

Figure S1 : Graph bars showing the percentage of samples analyzed in this study (dark grey bar) of the total number of samples tested for Mpox per year at INRB (light grey bar). The absolute number of samples included in the study or received at the INRB each year is displayed in the dark grey area and light grey area, respectively. The numbers displayed in the blue areas (upper graph) or yellow areas (lower graph) correspond to the total number of positive samples analyzed each year by PCR or serology, respectively
