# Supplementary material for: Use of Mpox Multiplex Serology in the Identification of Cases and Outbreak Investigations in the Democratic Republic of the Congo (DRC)
Source: Pathogens. 2023 Jul 7;12(7):916. doi: 10.3390/pathogens12070916 (PMC10385798; doi:10.3390/pathogens12070916)
Supplement: Supplementary file 1 [file pathogens-12-00916-s001.zip › supplementary_figures_review/Supplementary figures and tables.pdf]

pathogens

Supplementary figures

Figure S1 :workflow of the Mpox Surveillance Algorithm in DRC

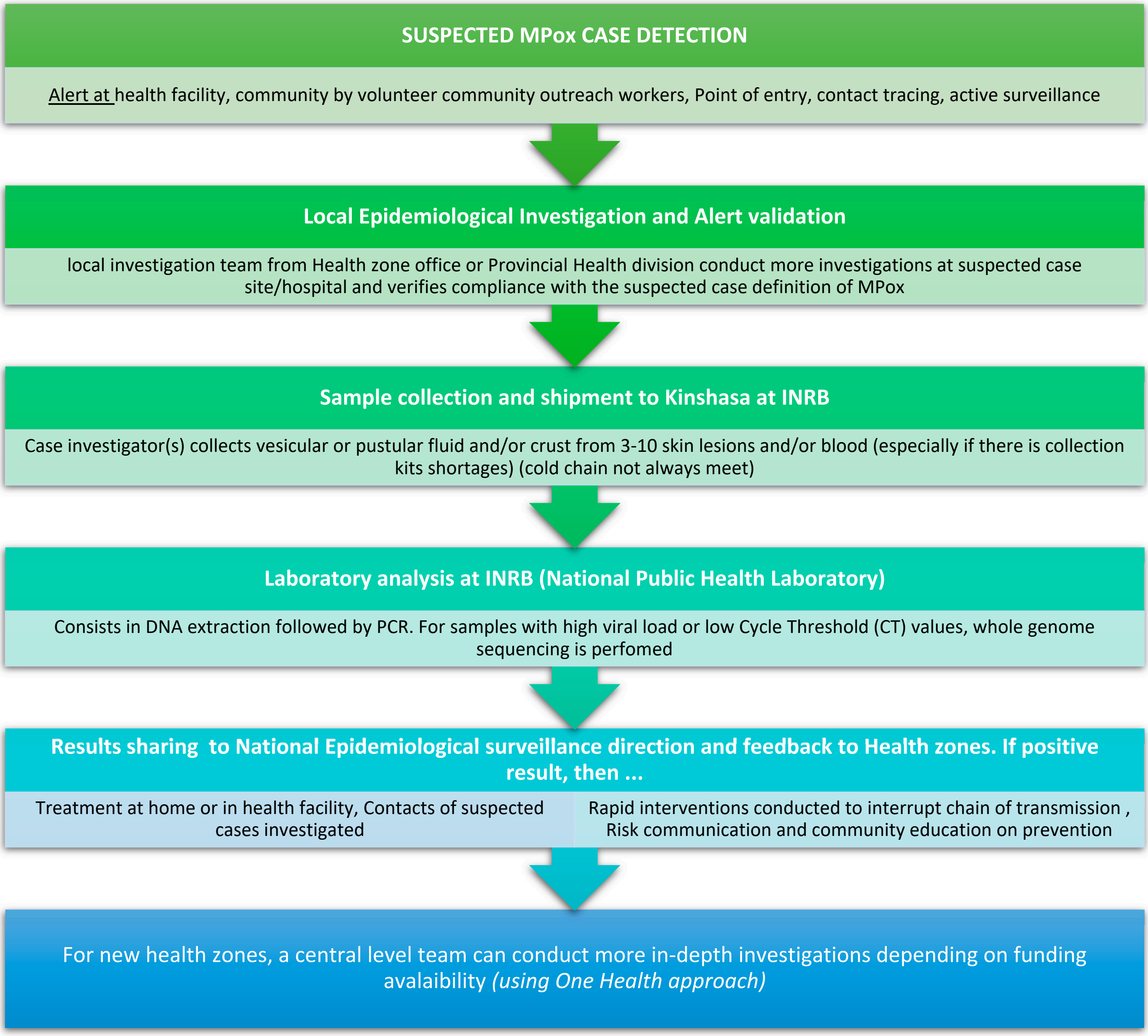

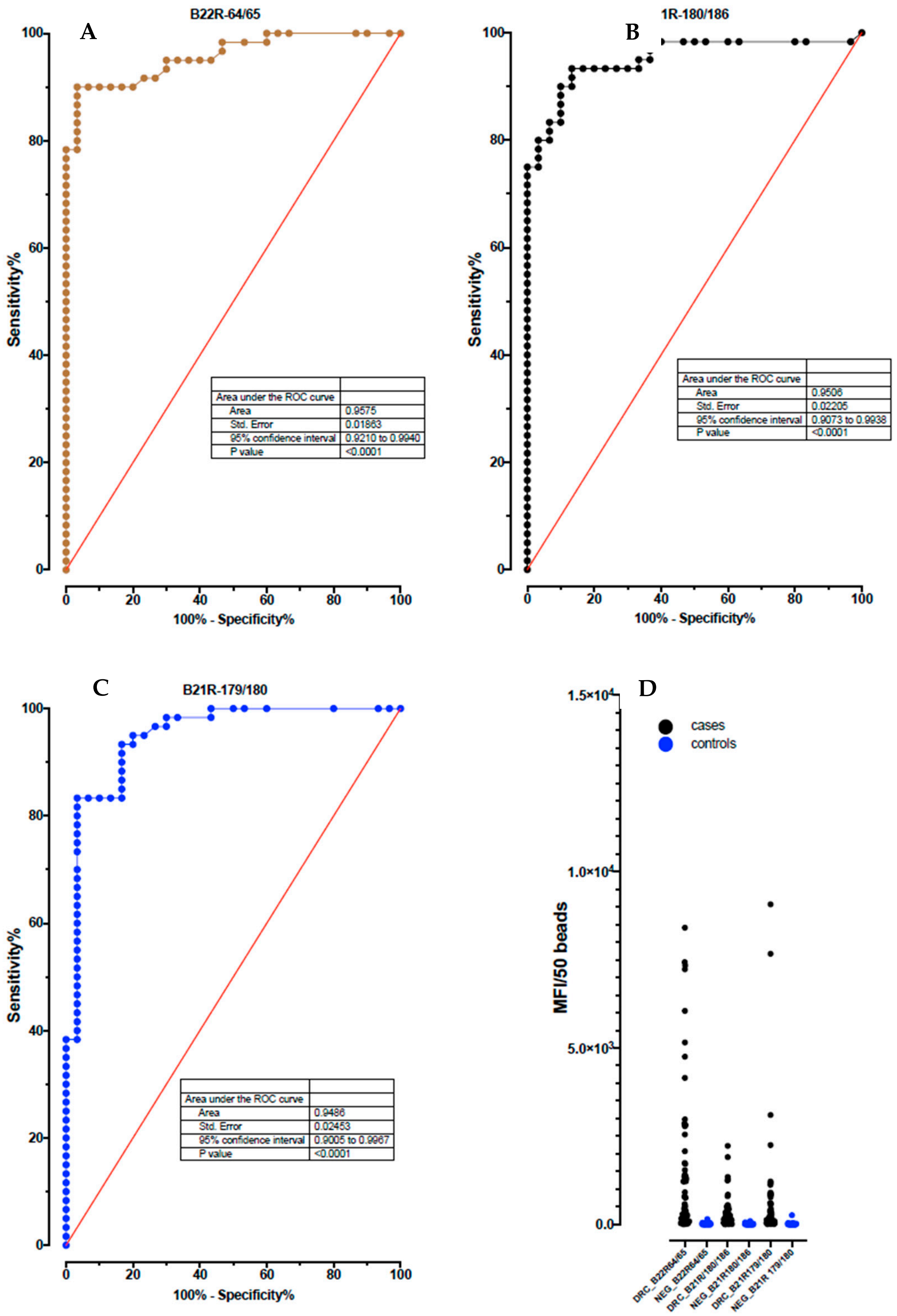

Figure S2 : Areas Under curve(AUC) of the Receiver Operating Characteristics (ROC) curves per peptid summarizing the performances of the assay for each peptid. Areas are respectively 0.9575, 0.9506 and 0.9486 for B22R.64/65 (A), B21R-180/186(B) and B21R-179/180(C). These peptids were kept for the multiplex serological assay. (D) The dotplot highlights Median Fluorecence Intensity (MFI)values per 50 beads for the panel of 90 blood samples including 60 Mpox cases (black dots) collected in the Democratic Republic of the Congo (DRC) and 30 control samples (blue dots) collected in France born after 1980). MFI are presented for each peptid and stratified by cases and controls samples. The ROC analysis was performed with GraphPad Prism 8.4.3 for MacOS.

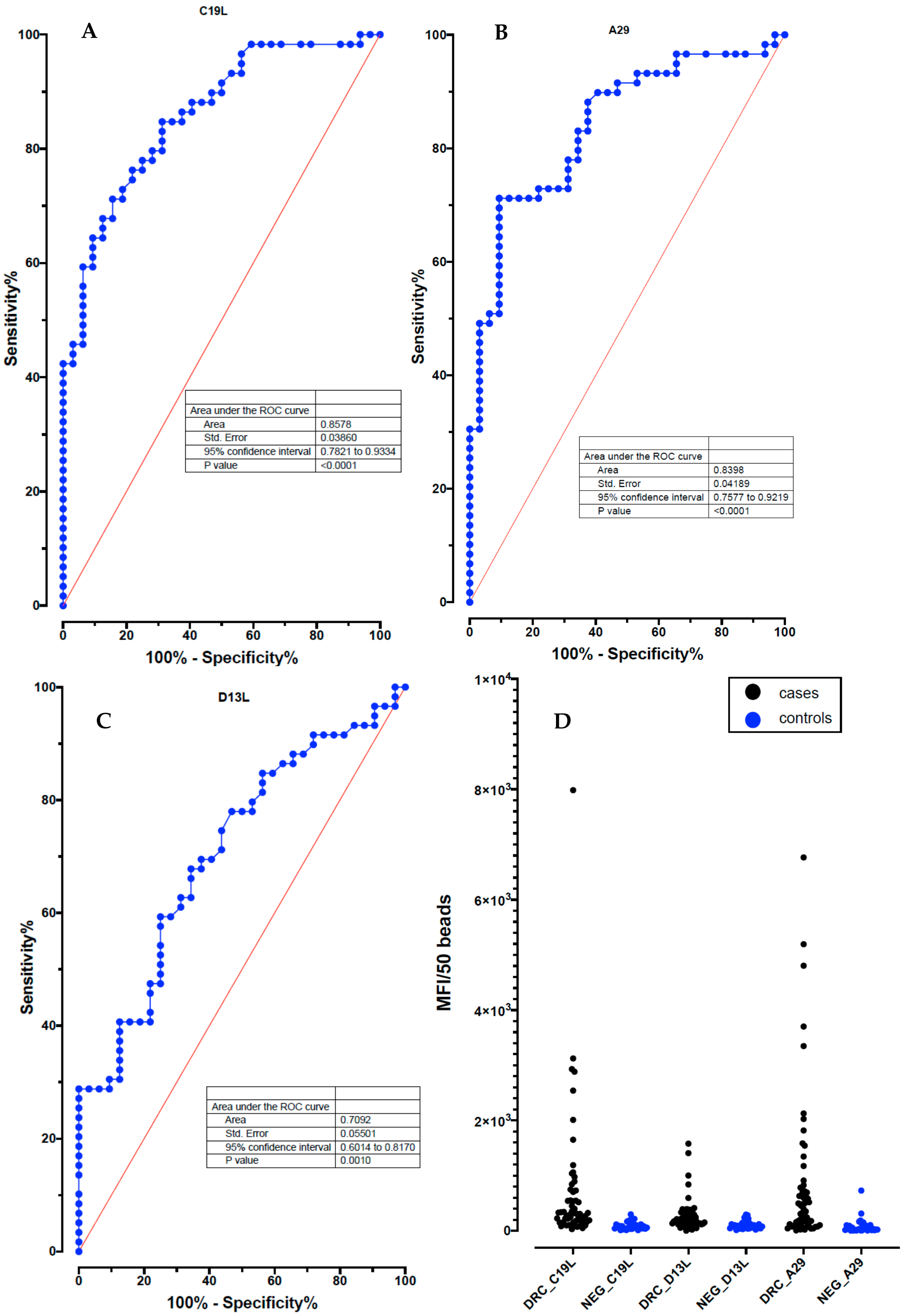

Figure S3 : Areas Under curve(AUC) of the Receiver Operating Characteristics (ROC) curves per protein summarizing their performances. AUC are 0.8578 for C19L(A), 0.8398 for A29(B), 0.7092 for D13L(C). Dots plots (D) summarize MFI/50 beads for the panel of 90 blood samples including 60 Mpox cases (black dots) collected in DRC and 30 control samples (blue dots) collected in France born after 1980. MFI are presented for each protein and stratified by cases and controls samples. The ROC analysis was performed with GraphPad Prism 8.4.3 for MacOS.

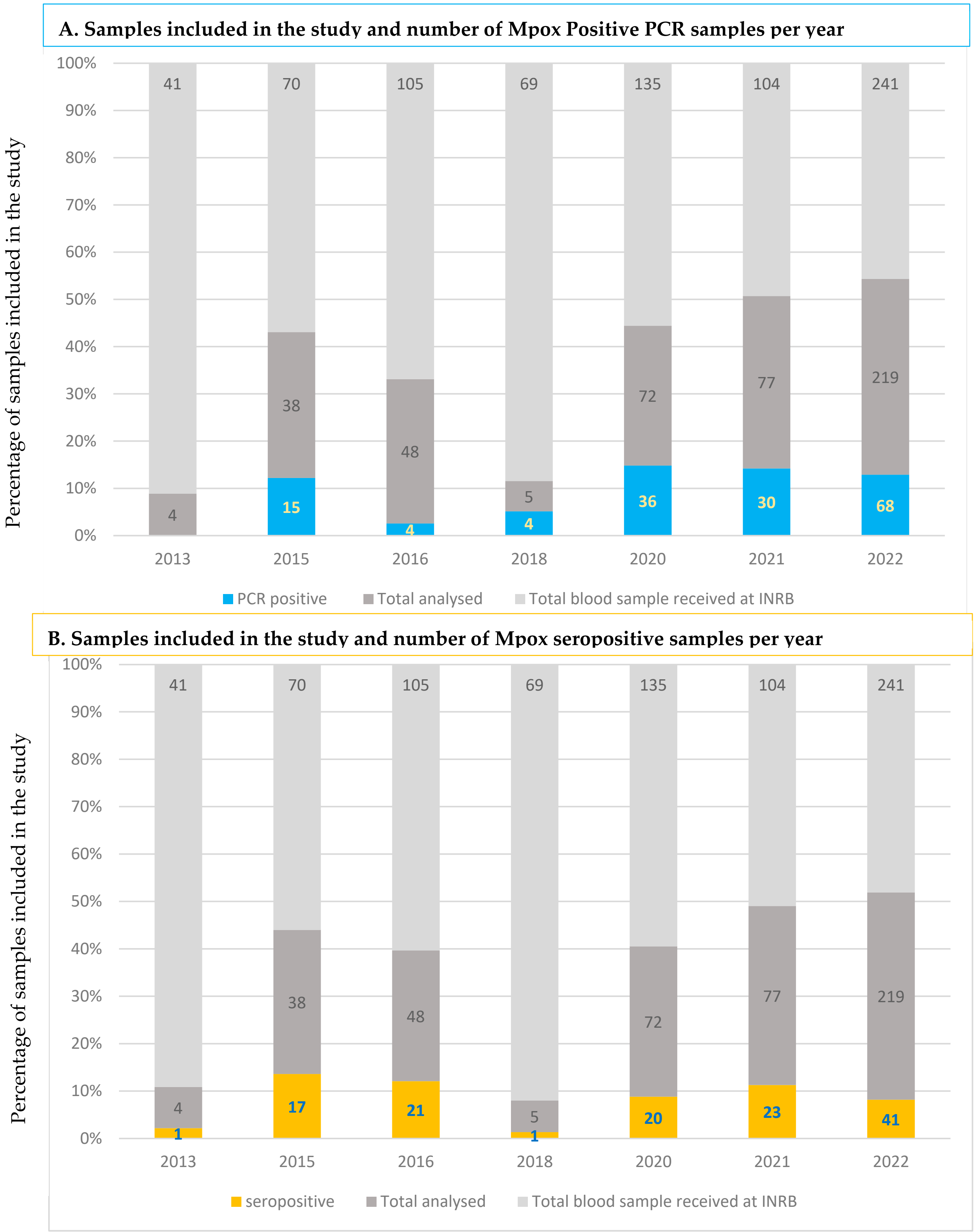

Figure S4 : Graph bars showing the percentage of samples analyzed in this study (dark grey bar) of the total number of samples tested for Mpox per year at INRB (light grey bar). The absolute number of samples included in the study or received at the INRB each year is displayed in the dark grey area and light grey area, respectively. The numbers displayed in the blue areas (upper graph) or yellow areas (lower graph) correspond to the total number of positive samples analyzed each year by PCR or serology, respectively

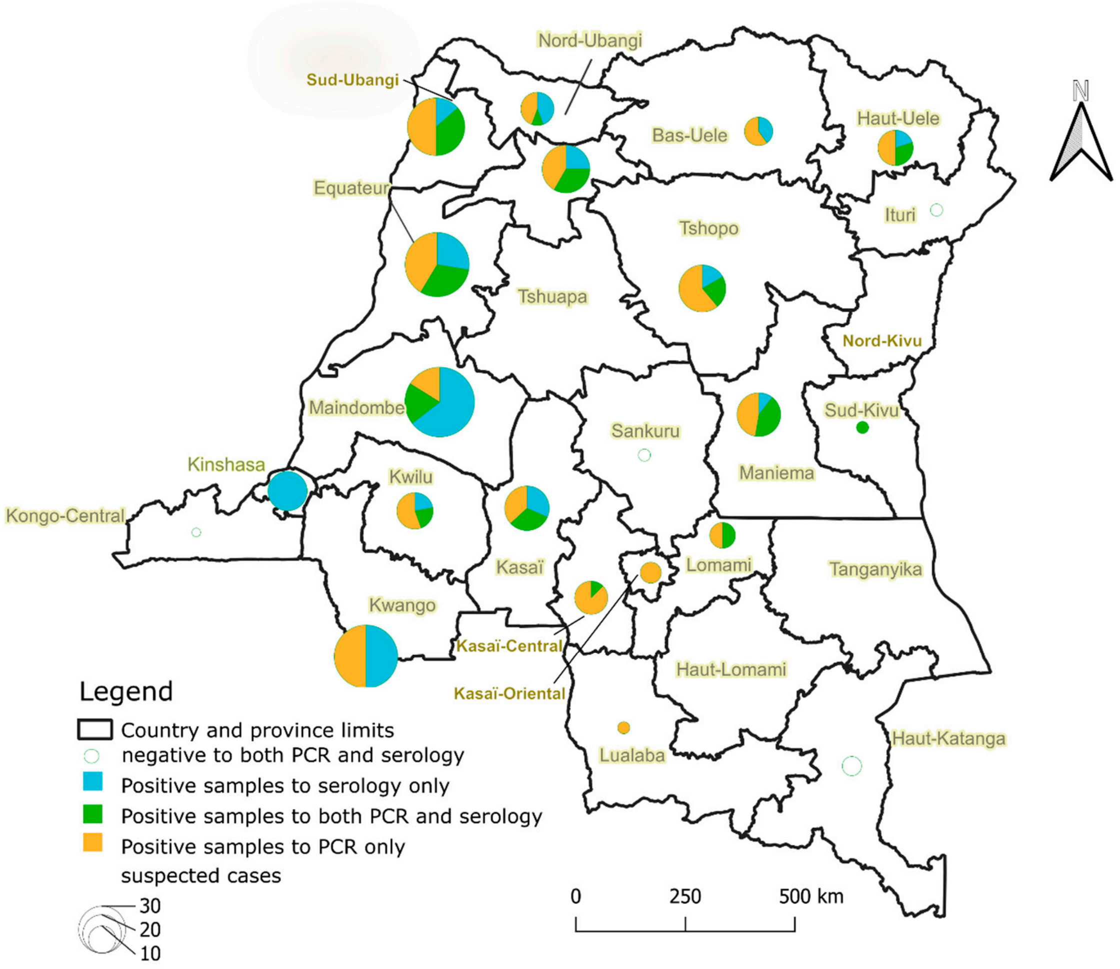

Figure S5 : Map of DRC Map showing the number of clinical Mpox suspected samples tested by province and proportions of samples confirmed by PCR only (yellow), serology only (blue) and samples positive by serology and PCR (green)

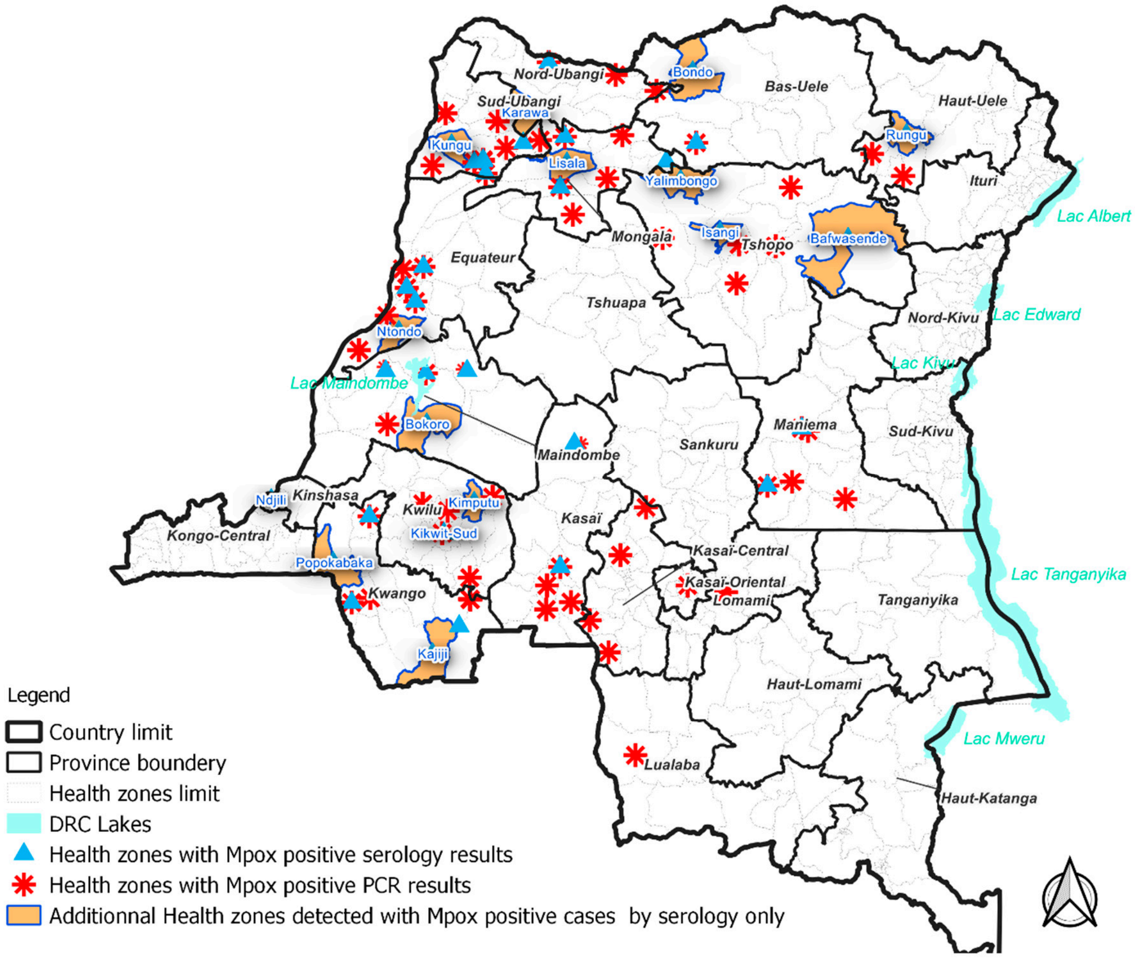

Figure S6 : DRC map representing health zones in which outbreaks were not detected by PCR with serology evidence of Mpox circulation(yellow) and health zones where originated Mpox positive cases by PCR(blue triangle) or Serology (red asterisk fill)

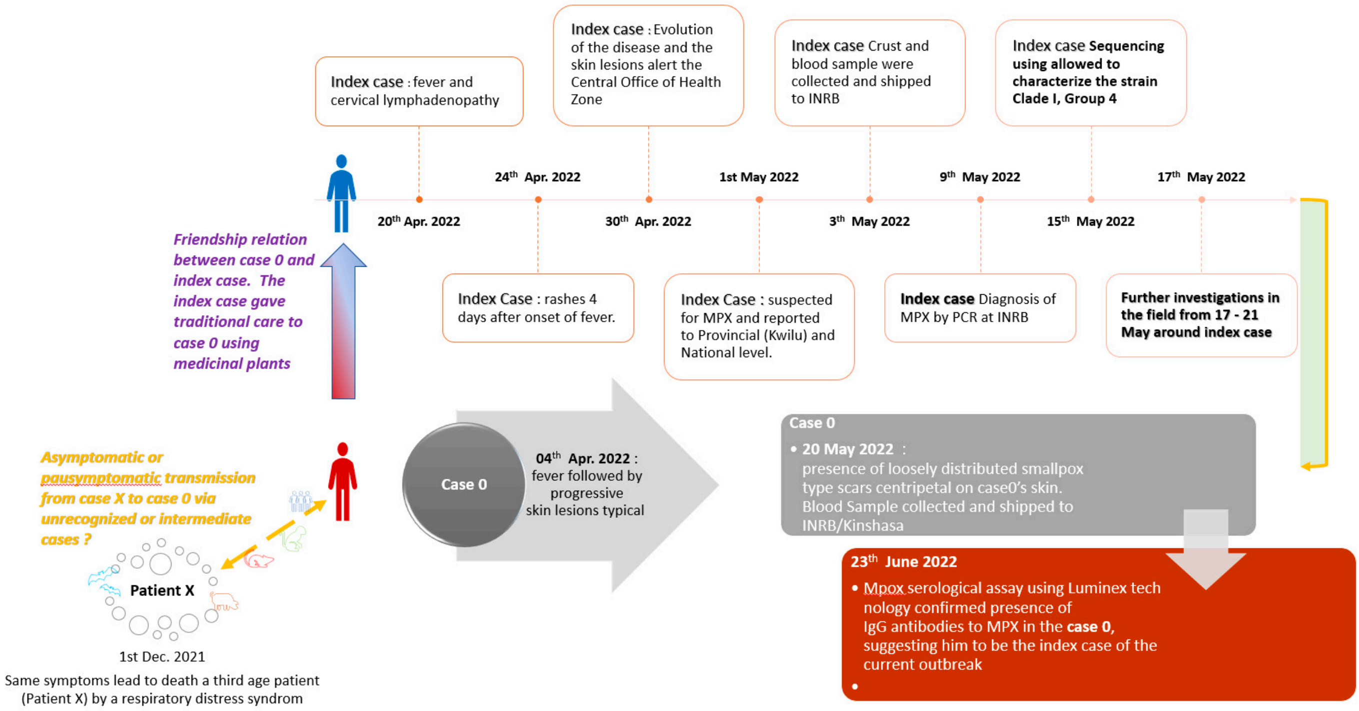

Figure S7 : Masimanimba Health zone in the Kwilu Province and main events around the first Mpox confirmed case

Table S1 : Characteristics of patients and samples per serological results

Variable	Mpox Serology positive N=124	Mpox Serology negative N=339	Total N=463	p
**Age[1], years**				
**median (IQR[#])**	14 (7-25)	12 (5-25)	13 (5-25)	0.3578
**Range**	<1-60 years	<1-76 years	<1-76 years	
**Sex[2],**				0.461
**Female**	55 (44.4%)	159 (46.9%)	214 (46.2%)	
**Male**	66 (53.2%)	163 (48.1%)	229 (49.5%)	
Missing	3 (2.4%)	17 (5.0%)	20 (4.3%)	
**Delay symptoms and sampling[3]**				
**Days (IQR)**	8 (5 – 11)	6 (4-9)	6 (4-10)	0.0016
**Range**	0–52 days	0 – 95 days	0 – 95 days	
**Delay sample shipment[4]**				
**Days (IQR)**	12 (8-18)	13 (8-21)	12 (8-20)	0.590
**Range**	3 – 66 days	0 – 57 days	0 – 66 days	

[1] Age is documented for 440 patients (120 Mpox seropositive and 320 negative)
[2] Sex is documented for 443 patients (121 Mpox seropositive and 322 negative), reported proportions in the manuscript don't take in account the missing data.
[3] Delay symptoms and sampling is documented for 378 patients (104 Mpox seropositive and 274 seronegative)
[4] Delay sample shipment is documented for 431 patients (121 Mpox seropositive and 310 seronegative)
[#]IQR; interquartile range
