# Supplementary figures and images for: Use of Mpox Multiplex Serology in the Identification of Cases and Outbreak Investigations in the Democratic Republic of the Congo (DRC)
Source: Pathogens. 2023 Jul 7;12(7):916. doi: 10.3390/pathogens12070916 (PMC10385798; doi:10.3390/pathogens12070916)

**Figure S1** :workflow of the Mpox Surveillance Algorithm in DRC

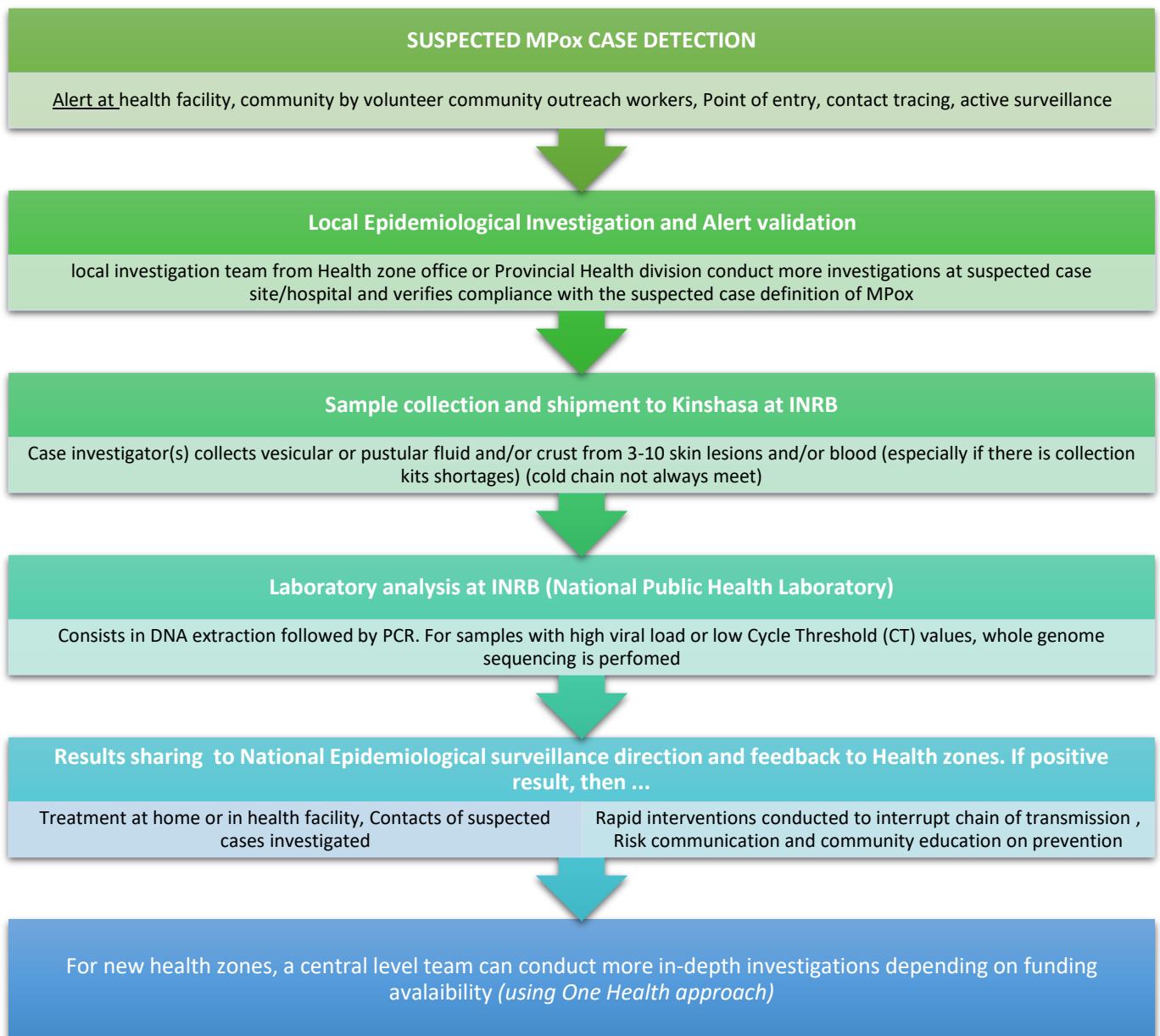

Supplement: Supplementary file 1 [file pathogens-12-00916-s001.zip › supplementary_figures_review/Figure S1.pdf]

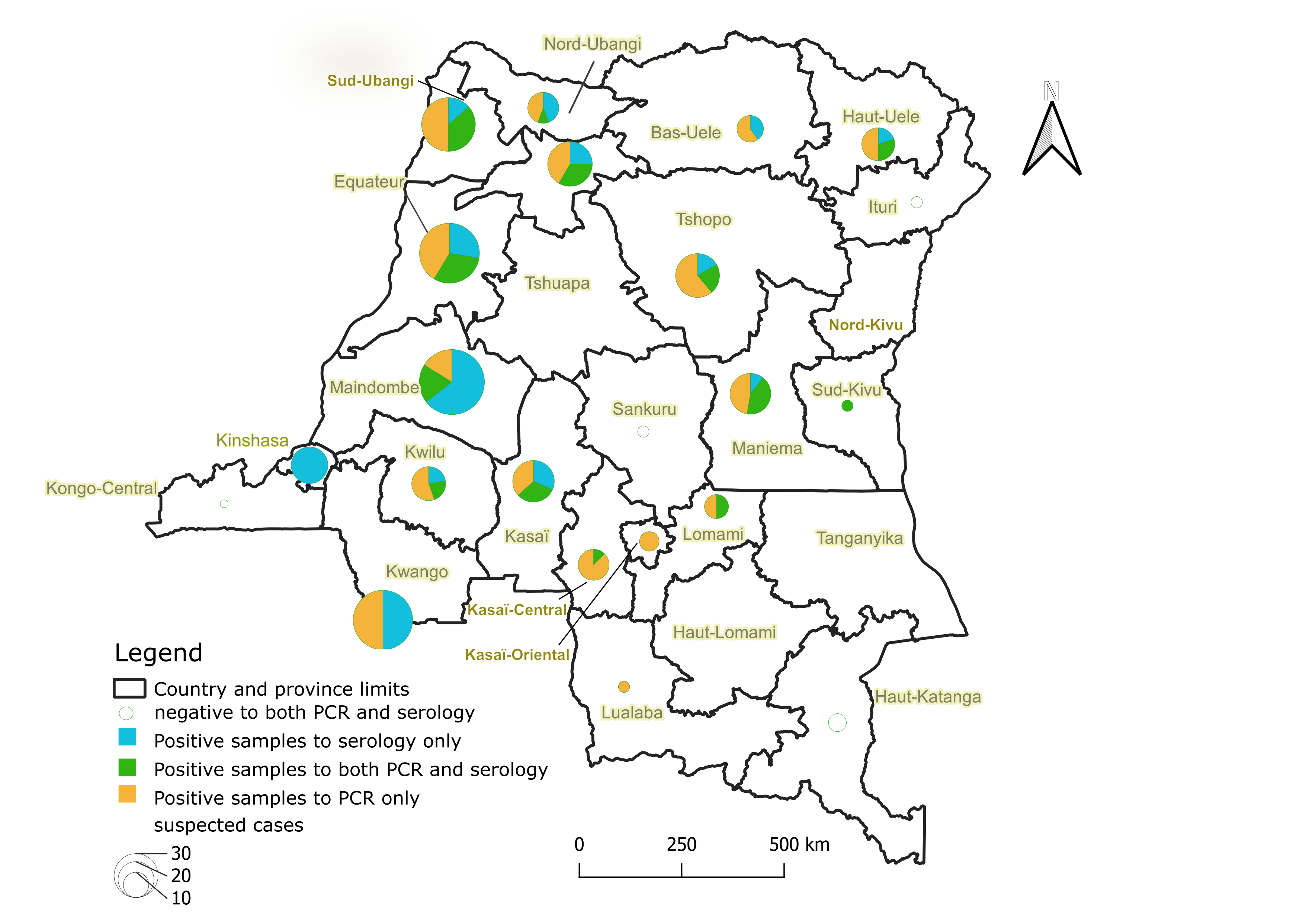

Supplement: Supplementary file 1 [file pathogens-12-00916-s001.zip › supplementary_figures_review/Figure S5.png]

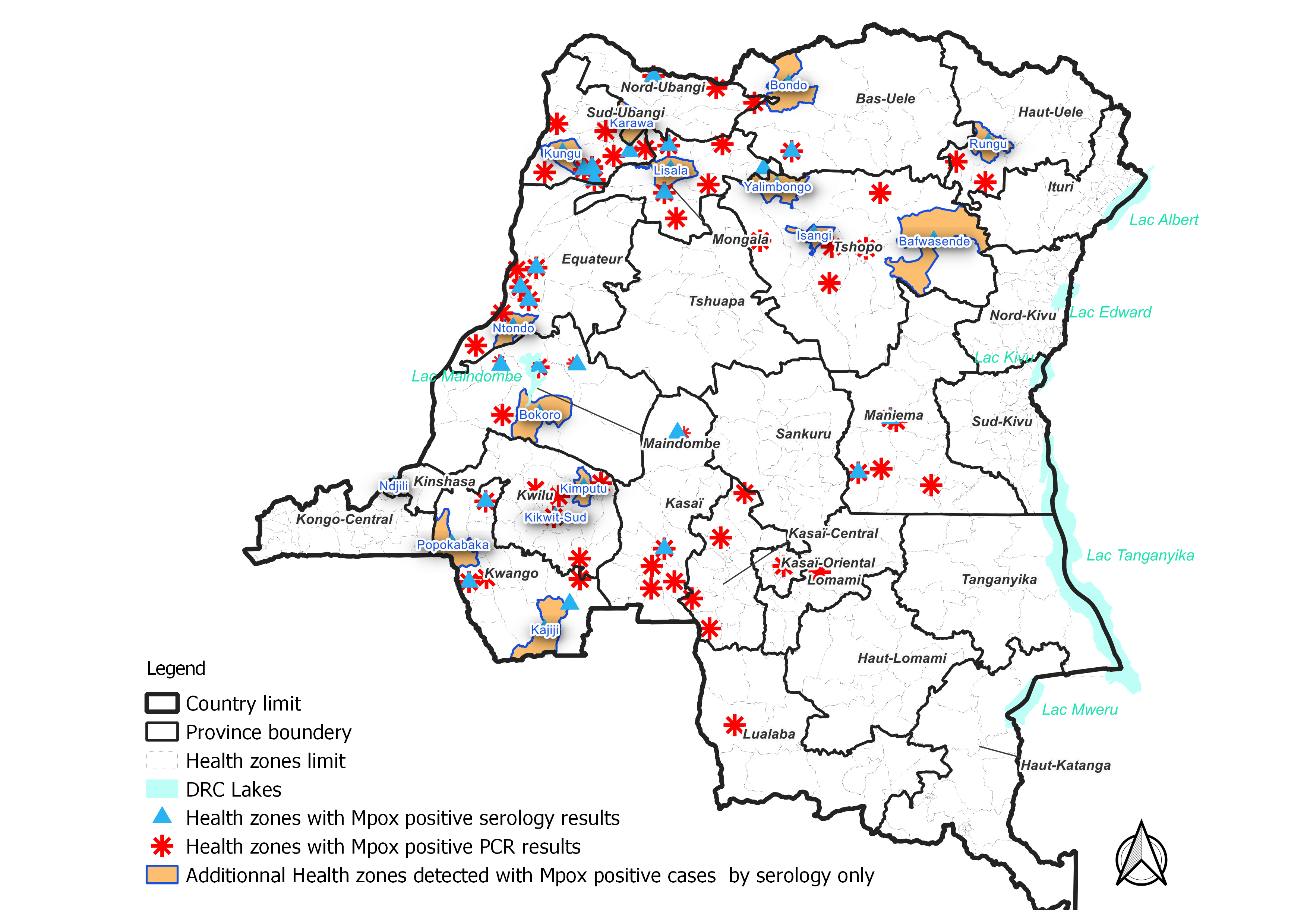

Supplement: Supplementary file 1 [file pathogens-12-00916-s001.zip › supplementary_figures_review/Figure S6.png]

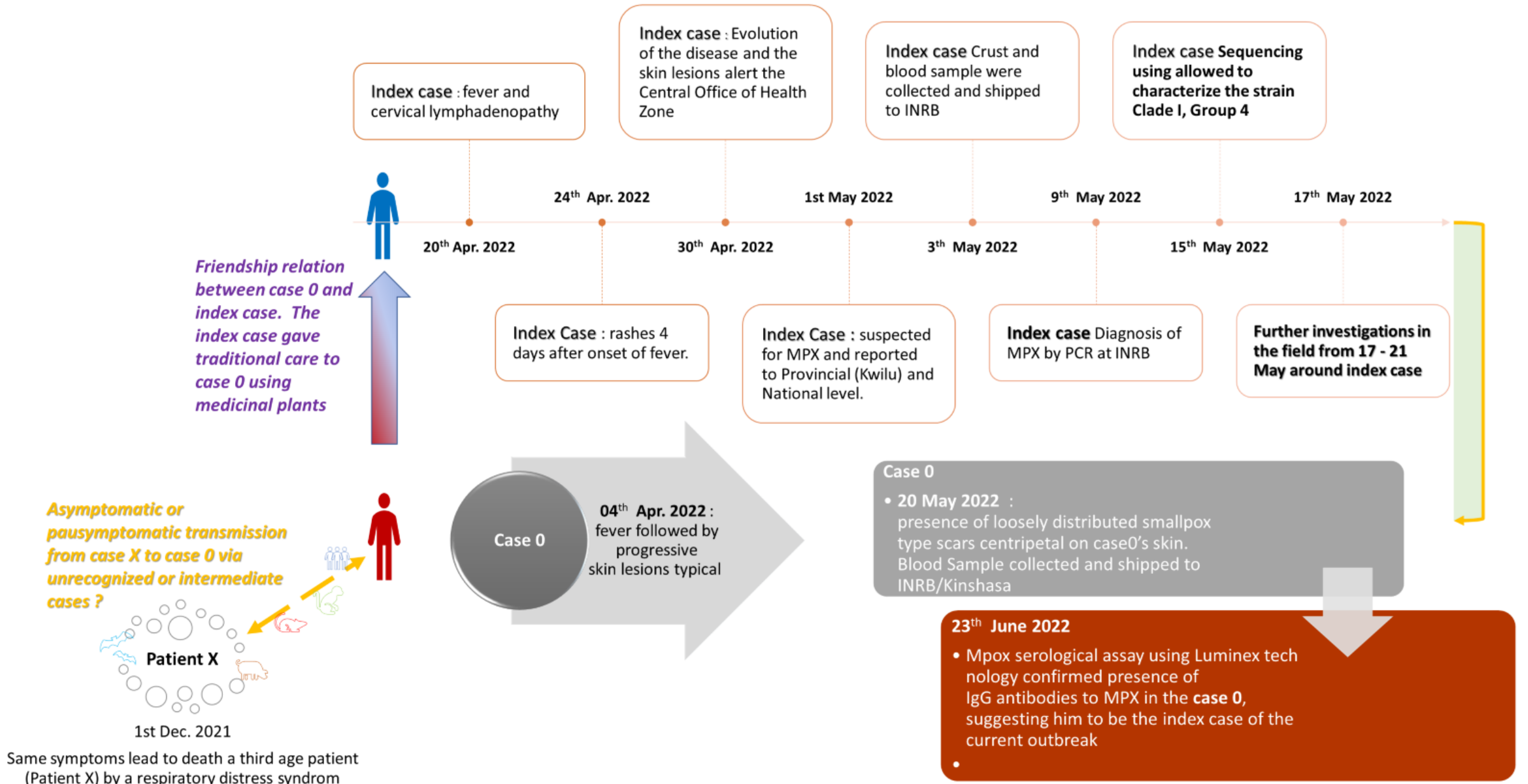

Supplement: Supplementary file 1 [file pathogens-12-00916-s001.zip › supplementary_figures_review/Figure S7.pdf]
